# *Curcuma longa* L. Extract and Photodynamic Therapy are Effective against *Candida* spp. and Do Not Show Toxicity *In Vivo*

**DOI:** 10.1155/2022/5837864

**Published:** 2022-07-01

**Authors:** Vanessa Marques Meccatti, Larissa de Souza Moura, Juliana Guerra Pinto, Juliana Ferreira-Strixino, Amjad Abu Hasna, Lívia Mara Alves Figueiredo-Godoi, Juliana Campos Junqueira, Maria Cristina Marcucci, Lucas de Paula Ramos, Claudio Antonio Talge Carvalho, Cesar Rogério Pucci, Luciane Dias de Oliveira

**Affiliations:** ^1^Department of Biosciences and Oral Diagnosis, Institute of Science and Technology, São Paulo State University (ICT-UNESP), São José dos Campos, São Paulo, Brazil; ^2^Photobiology Applied to Health (PHOTOBIOS)—University of Vale do Paraiba, Research and Development Institute. Av. Shishima Hifumi, 2911—São José dos Campos, São Paulo, Brazil; ^3^Department of Restorative Dentistry, Endodontics Division, Institute of Science and Technology, São Paulo State University (ICT-UNESP), São José dos Campos, São Paulo, Brazil; ^4^Department of Restorative Dentistry, Institute of Science and Technology, São Paulo State University (ICT-UNESP), São José dos Campos, São Paulo, Brazil

## Abstract

Radiotherapy induces a higher level of *Candida* spp. colonization, resulting in oral candidiasis. This study aimed to evaluate the phototransformation potential of the glycolic extract of *Curcuma longa (C. longa)*; the antifungal activity of *C. longa*, curcumin, and antifungal photodynamic therapy (aPDT) with blue light-emitting diodes “LED” on *Candida albicans* and *Candida tropicalis in vitro*; and the toxicity of *C. longa* and curcumin in *Galleria mellonella* model. In order to confirm the light absorption capacity of the *C. longa* extract, its phototransformation potential was evaluated. The antifungal effect of *C. longa*, curcumin, and aPDT was evaluated over *Candida* spp. Finally, the toxicity of *C. longa* and curcumin was evaluated on the *Galleria mellonella* model. The data were analyzed using the GraphPad Prism 5.0 software considering *α* = 5%. It was found that *C. longa*, curcumin, and aPDT using blue LED have an antifungal effect over *C. albicans* and *C. tropicalis*. The extract of *C. longa* 100 mg/mL and curcumin 200 *μ*g/mL do not show toxicity on* Galleria mellonella* model.

## 1. Introduction

Radiotherapy is a nonsurgical technique, and it is used to treat a variety of tumors by destroying cancer cells using X-rays, gamma rays, and other high-energy particles [1]. However, it causes oral candidiasis and radiation-induced oral mucositis [2] as it induces a higher level of *Candida* spp. colonization [3]. These yeasts have the ability to form biofilms and germ tubes, and they possess adhesion proteins and enzymes at the ends of hyphae that contribute to their diffusion into host tissues [4].

*Candida albicans* (*C. albicans*) is the main species responsible for causing oral candidiasis [5]. It has the ability to grow in anaerobiosis and has a role in endodontic and periodontal infections [6, 7]. Besides, *Candida tropicalis (C. tropicalis)*, the yeast that has a high capacity for adhesion and biofilm formation, has a role in fungal infections [8].

Currently, there are four main types of antifungal drugs including polyenes, azoles, allylamines, and echinocandins [9]. However, the incidence of fungal resistance to these drugs has increased [10] because of the excessive and uncontrolled use of antifungal drugs [11], in addition to the morphological and physiological changes provoked by radiotherapy; thus, the patients are becoming more susceptible to colonization by these yeasts [12], and only limited treatment options are effective for oral candidiasis [13].

Antimicrobial or antifungal photodynamic therapy (aPDT) consists of the combination of a photosensitizer with a light source that will produce reactive oxygen species [14] causing a cytotoxic effect on cancerous or microbial cells [15]. Some herbal medicines were tested and found effective over resistant microorganisms [16, 17] beside being biocompatible [18]. Turmeric, *Curcuma longa (C. longa)*, is a flowering plant of the ginger family, *Zingiberaceae* [19]. *C. longa* has curcumin, the herbal medicine, in which both have been widely studied for application in aPDT because of its molecular nature [20], effect over tumor cells [21, 22], and antifungal effect [23].

Diverse studies in the literature evaluated the effect of curcumin through aPDT over *C. albicans* [24, 25]. However, to the best of our knowledge, there is only one study that evaluated its effect over *C. tropicalis* [26], and there are no studies that evaluated the effect of glycolic extract of *C. longa* as a photosensitizer for aPDT over *Candida* spp. In addition, to advance toward the therapeutic use of the natural product, it is essential to study the toxicity of photosensitizers *in vivo*. Therefore, the aim of this study was to evaluate (I) the phototransformation potential of the glycolic extract of *C. longa*; (II) the antifungal activity of *C. longa*, curcumin, and aPDT with blue light-emitting diodes “LED” on *C. albicans* and *C. tropicalis in vitro*; (III) the toxicity of *C. longa* and curcumin in *Galleria mellonella* (*G. mellonella*) model. The null hypothesis was that *C. longa*, curcumin, and aPDT have no antifungal effect and are toxic.

## 2. Material and Methods

### 2.1. Fungus Strains and Plant Extract

The reference strains (ATCC, American Type Culture Collection) of *C. albicans* (ATCC 18804) and *C. tropicalis* (ATCC 13803), were used in the study and were obtained from the Laboratory of Microbiology and Immunology of the Institute of Science and Technology of São Paulo State University (ICT-UNESP).

The glycolic extract of *C. longa* (Seiva Brazilis, São Paulo, SP, Brazil) was obtained commercially at a concentration of 20% (200 mg/mL). The curcumin was produced by PDT Pharma (PDT Pharma, Cravinhos, SP, Brazil) and was provided by the Biophotonics Laboratory, Institute of Physics, University of São Paulo, São Carlos. According to the supplier's instructions, curcumin was initially diluted in a dimethylsulfoxide (DMSO) solution (at 1% of the final volume) and ethanol p.a. and then was kept as a stock solution. Posteriorly, it was diluted in phosphate buffer solution (PBS) to be tested.

### 2.2. Absorption Spectrum

To confirm the light absorption capacity of the *C. longa* extract and evaluate the possibility to be used as a photosensitizer, its phototransformation potential was evaluated. Aliquots of the plant extract at different dilutions were placed in cuvettes and subjected to analysis of optical characteristics. These were obtained through absorbance spectrum in a spectrophotometer (DeNovix DS-11, Wilmington, DE, United States) at the Nanosensors Laboratory of the Research and Development Institute of the University of Vale do Paraiba. This equipment is capable of performing the full-spectrum analysis in microvolumes, using the UV-Vis technique (190–840 nm).

### 2.3. Experimental Groups and Antifungal Photodynamic Therapy “aPDT” over *Candida* spp

- Group 1: Sterile saline solution (NaCl 0.9%) (negative control group).
- Group 2: Nystatin (100.000 UI/mL) (positive control group).
- Group 3: *C. longa* extract (100 mg/mL).
- Group 4: Curcumin (200 *μ*g/mL).
- Group 5: LED. The irradiation was performed using a prototype A LED-based device (Biotable Irrad/LED), with a wavelength of 450 ± 5 nm and a power of 3 W per LED.
- Group 6: aPDT: curcumin + blue LED.

Standardized inoculum of each strain was prepared of culture seeded on Sabouraud-Dextrose agar (SD, HiMedia, Mumbai, India) and in yeast extract-peptone-dextrose (YPD) broth after 24 h of incubation at 37°C. The inoculum was centrifuged at 5,000 rpm for 10 min (MPW 350-Med. Instruments, Poland), and the supernatant was discarded. The remaining deposit was resuspended in sterile saline solution (NaCl 0.9%) and homogenized in a Vortex for 10 s. This procedure was repeated twice to obtain a standardized inoculum of *C. albicans* and *C. tropicalis* by using a spectrophotometer (Micronal B-582, São Paulo, Brazil) at 530 nm and an optical density of 1.258 in a concentration of 1 × 10^8^ yeast cells per milliliter.

Aliquots of these standardized inoculums were distributed in 15 mL tubes according to the experimental groups and were centrifuged (5000 rpm for 10 min). The supernatant was discarded. The tubes corresponding to groups 1 and 5 received a saline solution, the group 2 was treated with nystatin, the group 3 were resuspended in the extract of *C. longa* L (100 mg/mL), and the groups 4 and 6 were resuspended in the extract of curcumin at the predetermined concentrations (200 *μ*g/mL). Then, they were homogenized in Vortex for 10 s and incubated at 37°C for 20 min, in the absence of light, considering this period as the preirradiation time (PIT). Then, the tubes were centrifuged again, the supernatant was discarded, and the remaining deposit of fungal cells from all groups was resuspended in saline solution. Aliquots of these final suspensions from the tubes were distributed in 24-well plates, where *n* = 10. The plates containing the groups 5 and 6 received irradiation of 10 J/cm^2^; 110 mW/cm^2^ for 91 s (1^st^ irradiance protocol) or 25 J/cm^2^, 110 mW/cm^2^ for 228 s (2^nd^ irradiance protocol). Groups 1, 2, 3, and 4 were kept in the dark for the same period of irradiation. Finally, serial dilutions were performed and 20 *μ*L of each dilution were seeded on Sabouraud-Dextrose agar using the drop technique. After 48 h of incubation, the colony-forming units per milliliter (CFU/mL) were counted.

### 2.4. Evaluation of Toxicity on Invertebrate Model *G. mellonella*

The concentrations used in the *in vitro* tests (100 mg/mL of the *C. longa* extract and 200 *μ*g/mL of curcumin) were inoculated in the last right proleg of larvae in order to verify toxicity. A group consisting of 15 larvae was selected for each product and, as a control, a group of larvae were inoculated with PBS in the proleg on the right side. A 10 *μ*L aliquot of each substance was inoculated with Hamilton syringes (Hamilton Inc., USA), and then the larvae were kept in Petri dishes at 37°C in the dark, without nutrition. After 24 hours of inoculations, the number of dead *G. mellonella* was recorded daily up to 168 hours (7 days) for analysis of the survival curve. Larvae were considered dead when they showed no movement after touching.

### 2.5. Statistical Analysis

The data normality was analyzed by using the following tests: Shapiro–Wilk, Kolmogorov–Smirnov, and D'Agostino and Pearson omnibus and for homogeneity using the BioEstat 5.0 software. Then, ANOVA and Tukey's test were used for parametric data, and the Kruskal–Wallis test and Dunn's test for nonparametric data. The data obtained in the survival curve of *G. mellonella* were analyzed by the log-rank method using the GraphPad Prism 5.0 software. In all tests, a significance level of 5% was considered.

## 3. Results

### 3.1. Absorption Spectrum

**Figure 1:**
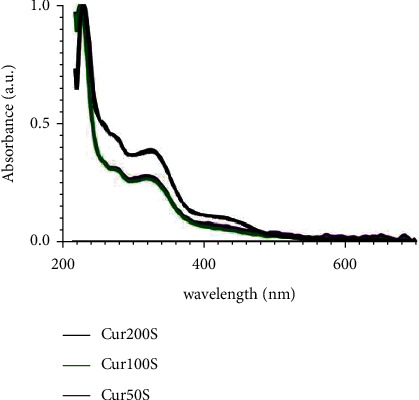
Absorption spectrum of the *C. longa* extract at different concentrations (200, 100, and 50 mg/mL) diluted in saline solution. Cur200S: curcumin extract of 200 mg/mL; Cur100S: curcumin extract of 100 mg/mL; and Cur50S: curcumin extract of 50 mg/mL.

The *C. longa* extract was not able to absorb light in the 450 nm wavelength range (Figure 1). The phototransformation potential test was carried out with different concentrations of the plant extract (200, 100, and 50 mg/mL) diluted in saline solution S. Therefore, it was tested as an herbal extract rather than a photosensitizer in the CFU/mL.

### 3.2. Antifungal Photodynamic Therapy “aPDT” over *Candida* spp

**Figure 2:**
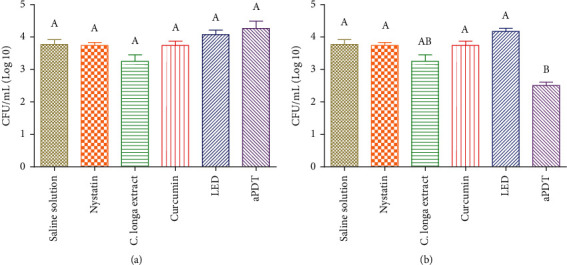
The colony-forming units CFU/mL of *C. albicans* for the 1^st^ and 2^nd^ irradiance protocol using curcumin as photosensitizer and *C. longa* as a plant extract. Uppercase letters (A, B, and AB) indicate a statistically significant difference: (a) 1^st^ irradiance protocol. (b) 2^nd^ irradiance protocol.

For the 1^st^ irradiation protocol (10 J/cm^2^ and 110 mW/cm^2^) against *C. albicans*, there was no statistically significant difference among all experimental groups (Figure 2(a)). For the 2^nd^ irradiation protocol (25 J/cm^2^·and 110 mW/cm^2^) against *C. albicans*, the *C. longa* extract was able to promote almost 1 log reduction and had no statistically significant difference with the aPDT group (LED + curcumin). However, the aPDT group was more effective than the *C. albicans* group and it had a statistically significant difference from all other groups (Figure 2(b)).

**Figure 3:**
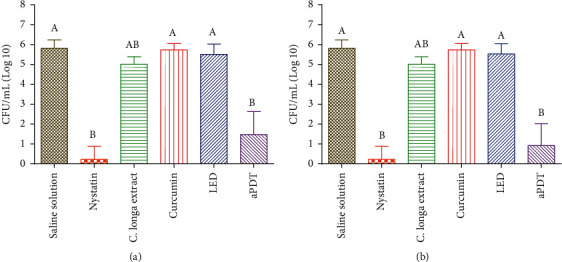
The colony-forming units CFU/mL of *C. tropicalis* for the 1^st^ and 2^nd^ irradiance protocol using curcumin as photosensitizer and *C. longa* as a plant extract. Uppercase letters (A, B, and AB) indicate a statistically significant difference. (a) 1^st^ irradiance protocol. (b) 2^nd^ irradiance protocol.

For the 1^st^ irradiation protocol (10 J/cm^2^ and 110 mW/cm^2^) and the 2^nd^ irradiation protocol (25 J/cm^2^·e 110 mW/cm^2^) against *C. tropicalis*, the groups of nystatin and aPDT were effective over the yeast and had a statistically significant difference of all groups except the *C. longa* group. In the 1^st^ irradiation protocol, it was observed that the aPDT group was able to promote almost 4 log reduction and in the 2^nd^ irradiation protocol, it was able to promote almost 5 log reduction. The *C. longa* extract applied alone was able to promote almost 1 log reduction and when compared to the aPDT or nystatin group, it did not show any difference (Figures 3(a) and 3(b)).

### 3.3. Evaluation of Toxicity of *C. longa* and Curcumin on Invertebrate Model *G. mellonella*

**Figure 4:**
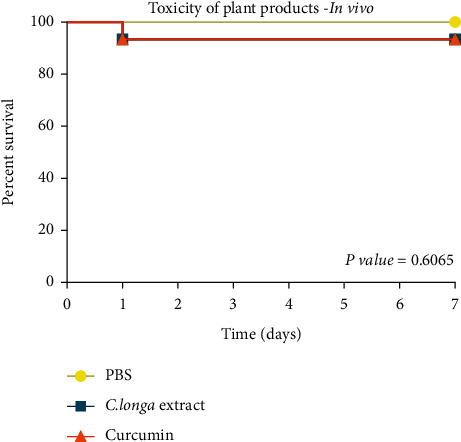
Survival curve of *G. mellonella* larvae after inoculation of the glycolic extract of *C. longa* (100 mg/mL) and curcumin (200 *μ*g/mL) compared to control (PBS) (*n* = 15, log-rank test, Mantel–Cox).

The toxicity analysis of the *C. longa extract* (100 mg/mL) and curcumin (200 *μ*g/mL) was also performed by testing the survival curve of larvae evaluated for 7 days after inoculation.  Figure 4 shows the statistical similarity between the groups of herbal and control products (PBS) with (*P*=0.60). The products derived from *C. longa* do not show toxicity in the invertebrate model.

## 4. Discussion

PDT is a promising alternative therapy that has emerged in dentistry for the treatment of oral cavity infections [15, 27, 28]. This study was elaborated to evaluate the antifungal effect of *C. longa*, curcumin (as photosensitizer or herbal extract), and aPDT over *C. albicans* and *C. tropicalis*. The null hypothesis was rejected.

An important parameter to assess the photodynamic effectiveness of a photosensitizer is the analysis of the number of absorbed photons needed to cause the transformation of a fixed amount of sensitizer. To confirm the light absorption capacity of *C. longa* extract, its phototransformation potential was evaluated. It was found that the *C. longa* extract was not able to absorb light in the 450 nm wavelength range (Figure 1). However, different results were reported in the literature found that the *C. longa* extract has a spectrum similar to the phytochemical curcumin isolated in the range from 400 to 440 nm [29].

In this study, it was opted to apply curcumin but not *C. longa* as a photosensitizer, based on the literature, the molecular nature and potential of absorption of curcumin light are well described [30, 31]. According to the literature, curcumin absorbs light within the entire visible spectrum of blue, in the range from 300 to 500 nm [32] and its maximum absorbance was verified at an average of 418 nm depending on the solvents used [33].

However, over *C. albicans*, curcumin promoted a reduction in the CFU/mL count when using the second irradiation protocol (110 mW/cm^2^; 25 J/cm^2^ for 228 s) with a PIT of 20 min. Similar results were reported previously, in which a decrease in the viability of planktonic cells and *C. albicans* biofilm (22 mW/cm^2^, 5.28 J/cm^2^, 455 nm, 240 s) using curcumin as a photosensitizer with PIT of 20 min. Curcumin, as a lipophilic molecule, interacts directly with the membrane and membrane proteins, which explains why prolonged preirradiation times are not necessary [34]. The study of Ma et al. evaluated the use of PDT against ATCC and clinical strains of *C. albicans*, comparing different irradiation protocols, and found a time-dependent decrease in cell viability, as the longer the irradiation time, the higher the light dose used and consequently, greater inhibition of *C. albicans* [35]. In the present study, it was also observed a greater reduction in CFU/mL when the protocol with a long time and light dose was used, especially in *C. tropicalis*.

In the present study, the *C. longa* extract was effective alone over *C. albicans*. In the literature, fifteen plant extracts were evaluated for antifungal activity on clinical strains of *C. albicans* and the extract of *C. longa* was among the five herbal medicines that showed the best inhibitory effect and potential for controlling the growth of this yeast [36]. The turmeric extract was also evaluated for its potential to mediate PDT on planktonic cultures and *Enterococcus faecalis* biofilm and for toxicity on fibroblast cultures. The action of the herbal medicine as a photosensitizer was able to promote significant microbial reduction and produce cell viability similar to the methylene blue dye [29].

To advance toward the therapeutic use of the natural product, it is essential to study the toxicity of photosensitizers *in vivo. G. mellonella* is a widely used animal model that has interesting applicability. Investigations of the toxicity of substances in this *in vivo* model are of great importance, as they will enable the reduction of vertebrate animals for the same purpose.

The *C. longa* extract has antifungal activity and low *in vivo* systemic toxicity as it was able to promote a higher survival rate for the larvae in the study. In the present study, the antifungal potential of the plant extracts and the safety of use *in vivo* were also verified, since the extract of the *C. longa* plant and its isolated phytocompound curcumin did not affect the survival of the larvae, remaining similar to the control group that was inoculated only PBS being *P* > 0.05. The present study elucidated important findings regarding the application of plant products in PDT against *Candida* spp. since the effect of this therapy was equal to or even better than nystatin.

## 5. Conclusions

- The isolated *C. longa* extract and photodynamic therapy using curcumin as a photosensitizer with blue LED therapy have an antifungal effect over *C. albicans* and *C. tropicalis*;
- *C. longa* extract 100 mg/mL and curcumin 200 *μ*g/mL do not show toxicity for the invertebrate model *G. mellonella*.

## Data Availability

The data used to support the findings of this study are available from the corresponding author upon request.
